# Supplementary material for: Quantitative Risk Assessment of Bacillus cereus Growth during the Warming of Thawed Pasteurized Human Banked Milk Using a Predictive Mathematical Model
Source: Foods. 2022 Apr 2;11(7):1037. doi: 10.3390/foods11071037 (PMC8997632; doi:10.3390/foods11071037)
Supplement: Supplementary file 1 [file foods-11-01037-s001.zip › Supplemetary tables.pdf]

Table S1. Prediction of *B. cereus* growth ine the thawed PBM for parameters of bottle No. 1

Time (hours)	Predicted microbial counts CFU/mL	Prediction uncertainty interval (CFU/mL) Lower limit	Upper limit
0	1.30×10^2	1.30×10^2	1.30×10^2
0.5	1.43×10^2	1.36×10^2	1.68×10^2
1	1.87×10^2	1.48×10^2	4.56×10^2
1.5	3.22×10^2	1.73×10^2	3.11×10^3
2	7.62×10^2	2.18×10^2	3.38×10^4
2.5	2.39×10^3	3.03×10^2	3.22×10^5
3	8.93×10^3	4.68×10^2	1.94×10^6

Table S2. Prediction of *B. cereus* growth ine the thawed PBM for parameters of bottle No. 2

Time (hours)	Predicted microbial counts CFU/mL	Prediction uncertainty interval (CFU/mL) Lower limit	Upper limit
0	2.00×10^2	2.00×10^2	2.00×10^2
0.5	2.05×10^2	2.01×10^2	2.28×10^2
1	2.24×10^2	2.04×10^2	5.89×10^2
1.5	2.89×10^2	2.10×10^2	5.77×10^3
2	4.89×10^2	2.21×10^2	9.95×10^4
2.5	1.13×10^3	2.40×10^2	1.15×10^6
3	3.45×10^3	2.72×10^2	6.37×10^6

Table S3. Prediction of *B. cereus* growth ine the thawed PBM for parameters of bottle No. 3

Time (hours)	Predicted microbial counts CFU/mL	Prediction uncertainty interval (CFU/mL) Lower limit	Upper limit
0	2.00×10^2	2.00×10^2	2.00×10^2
0.5	2.04×10^2	2.01×10^2	2.26×10^2
1	2.18×10^2	2.03×10^2	5.62×10^2
1.5	2.64×10^2	2.07×10^2	5.18×10^3
2	3.93×10^2	2.13×10^2	8.69×10^4
2.5	7.55×10^2	2.24×10^2	1.02×10^6
3	1.87×10^3	2.41×10^2	5.77×10^6

Table S4. Prediction of *B. cereus* growth during warming of thawed PBM for different initial post-pasteurization CFU concentrations.

Initial concentration (CFU/mL)	Concentration after 1 hour (CFU/mL)			Concentration after 2 hours (CFU/mL)			Concentration after 3 hours (CFU/mL)		
CFU count	CFU count	Lower uncertainty limit	Upper uncertainty limit	CFU count	Lower uncertainty limit	Upper uncertainty limit	CFU count	Lower uncertainty limit	Upper uncertainty limit
1.0	1.5	1.2	3.6	6.0	1.7	2.6×10^2	69.0	3.7	1.5×10^4
5.0	7.3	5.8	18.0	30.0	8.5	1.3×10^3	3.5×10^2	18.0	7.7×10^4
6.0	8.8	6.9	21.0	36.0	10.0	1.5×10^3	4.1×10^2	22.0	9.3×10^4
10.0	15.0	12.0	36.0	60.0	17.0	2.6×10^3	6.9×10^2	37.0	1.5×10^5
11.0	16.0	13.0	39.0	66.0	19.0	2.8×10^3	7.6×10^2	40.0	1.7×10^5
15.0	22.0	17.0	53.0	89.0	25.0	3.8×10^3	1.0×10^3	55.0	2.3×10^5
16.0	23.0	18.0	57.0	95.0	27.0	4.1×10^3	1.1×10^3	59.0	2.5×10^5
20.0	29.0	23.0	71.0	1.2×10^2	34.0	5.1×10^3	1.4×10^3	73.0	3.1×10^5
21.0	31.0	24.0	75.0	1.3×10^2	36.0	5.4×10^3	1.4×10^3	77.0	3.2×10^5
25.0	37.0	29.0	89.0	1.5×10^2	42.0	6.4×10^3	1.7×10^3	92.0	3.9×10^5
26.0	38.0	30.0	93.0	1.5×10^2	44.0	6.7×10^3	1.8×10^3	95.0	4.0×10^5
30.0	44.0	35.0	1.1×10^2	1.8×10^2	51.0	7.7×10^3	2.1×10^3	1.1×10^2	4.6×10^5
31.0	45.0	36.0	1.1×10^2	1.8×10^2	53.0	7.9×10^3	2.1×10^3	1.1×10^2	4.8×10^5
35.0	51.0	40.0	1.2×10^2	2.1×10^2	59.0	9.0×10^3	2.4×10^3	1.3×10^2	5.4×10^5
36.0	53.0	41.0	1.3×10^2	2.1×10^2	61.0	9.2×10^3	2.5×10^3	1.3×10^2	5.6×10^5
40.0	58.0	46.0	1.4×10^2	2.4×10^2	68.0	1.0×10^4	2.8×10^3	1.5×10^2	6.2×10^5
41.0	60.0	47.0	1.5×10^2	2.4×10^2	70.0	1.1×10^4	2.8×10^3	1.5×10^2	6.3×10^5
45.0	66.0	52.0	1.6×10^2	2.7×10^2	76.0	1.2×10^4	3.1×10^3	1.6×10^2	7.0×10^5
46.0	67.0	53.0	1.6×10^2	2.7×10^2	78.0	1.2×10^4	3.2×10^3	1.7×10^2	7.1×10^5
50.0	73.0	58.0	1.8×10^2	3.0×10^2	85.0	1.3×10^4	3.5×10^3	1.8×10^2	7.7×10^5
51.0	75.0	59.0	1.8×10^2	3.0×10^2	87.0	1.3×10^4	3.5×10^3	1.9×10^2	7.9×10^5
99.0	1.4×10^2	1.1×10^2	3.5×10^2	5.9×10^2	1.7×10^2	2.5×10^4	6.8×10^3	3.6×10^2	1.5×10^6
1.0×10^2	1.5×10^2	1.2×10^2	3.6×10^2	6.0×10^2	1.7×10^2	2.6×10^4	6.9×10^3	3.7×10^2	1.5×10^6
