# Supplementary figures and images for: Quantitative Risk Assessment of Bacillus cereus Growth during the Warming of Thawed Pasteurized Human Banked Milk Using a Predictive Mathematical Model
Source: Foods. 2022 Apr 2;11(7):1037. doi: 10.3390/foods11071037 (PMC8997632; doi:10.3390/foods11071037)

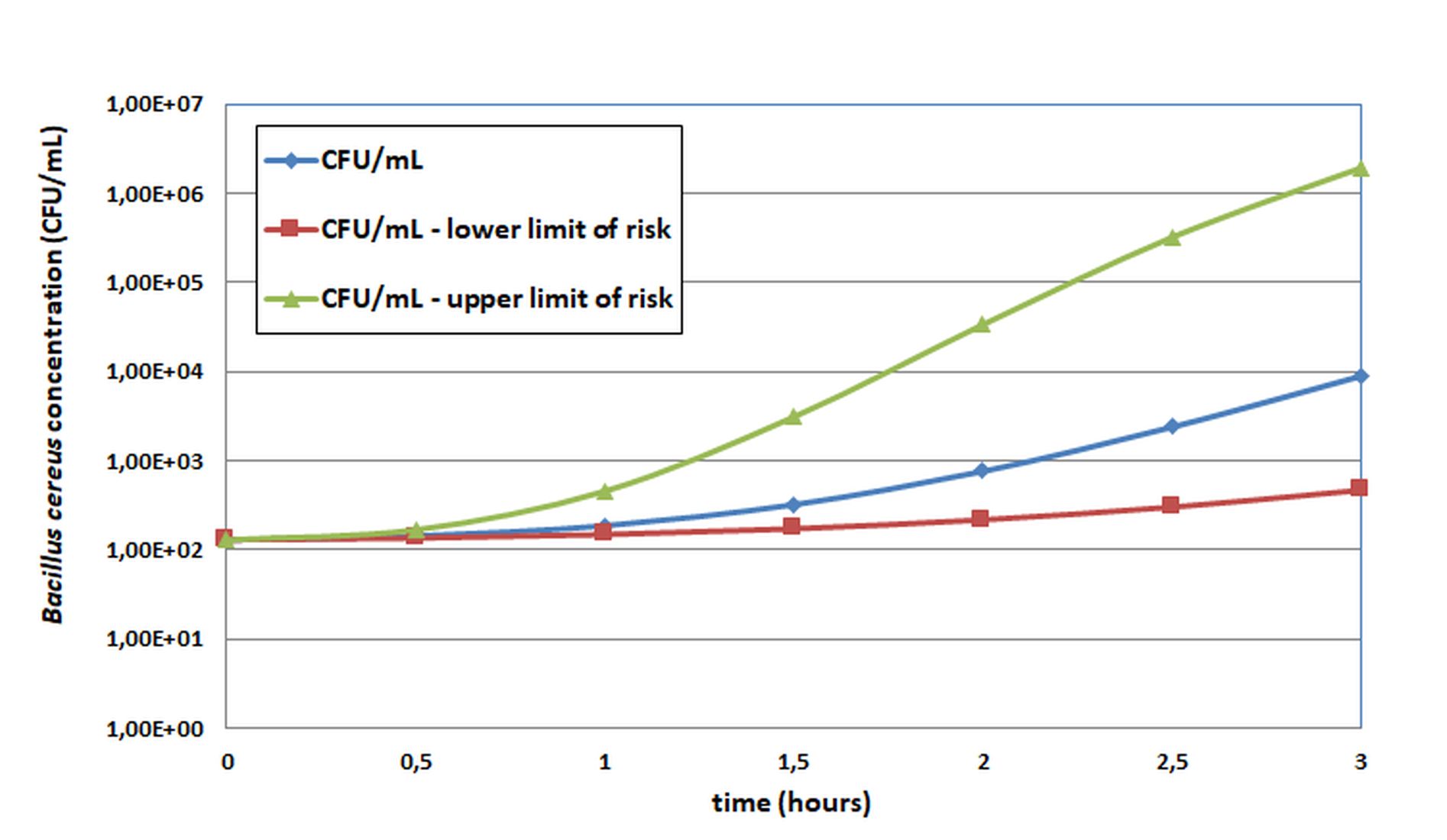

Supplement: Supplementary file 1 [file foods-11-01037-s001.zip › Figure S1_supplementary data.jpg]

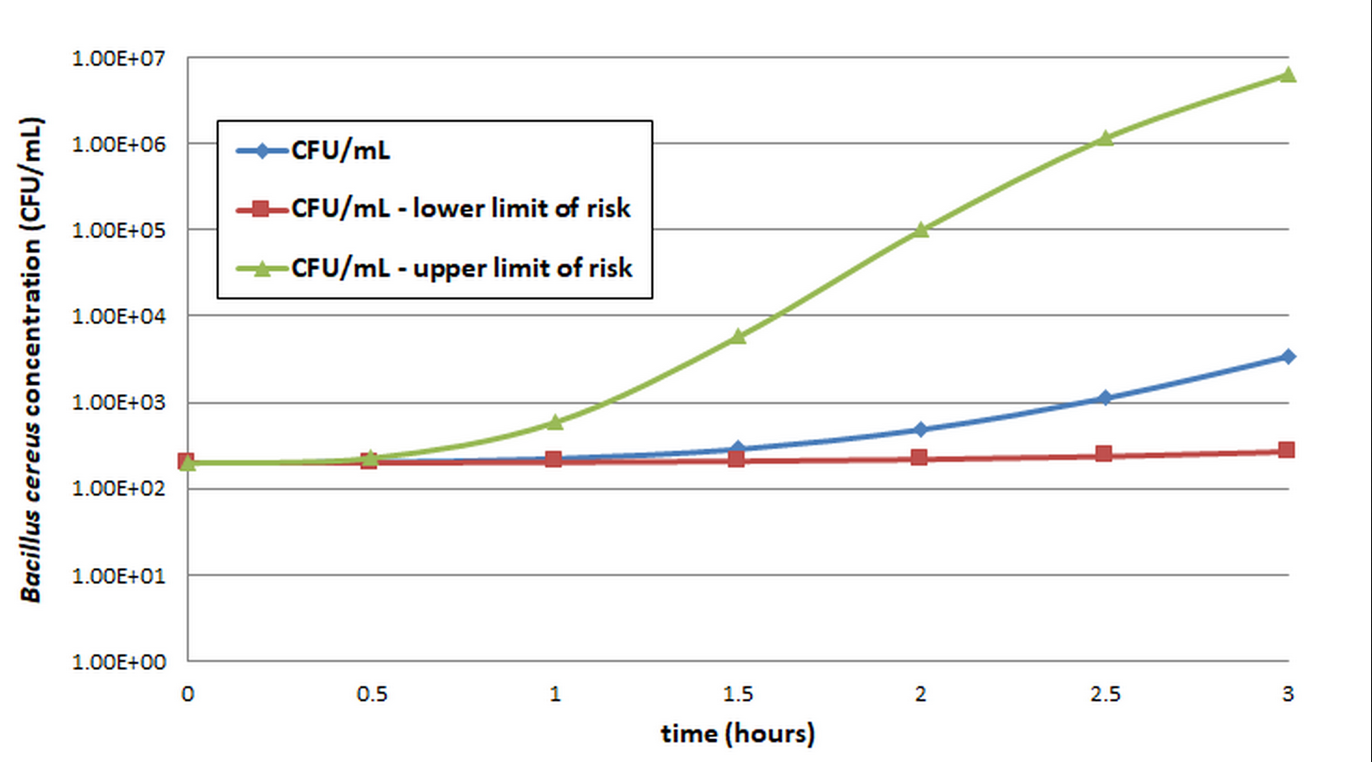

Supplement: Supplementary file 1 [file foods-11-01037-s001.zip › Figure S2_supplementary data.png]

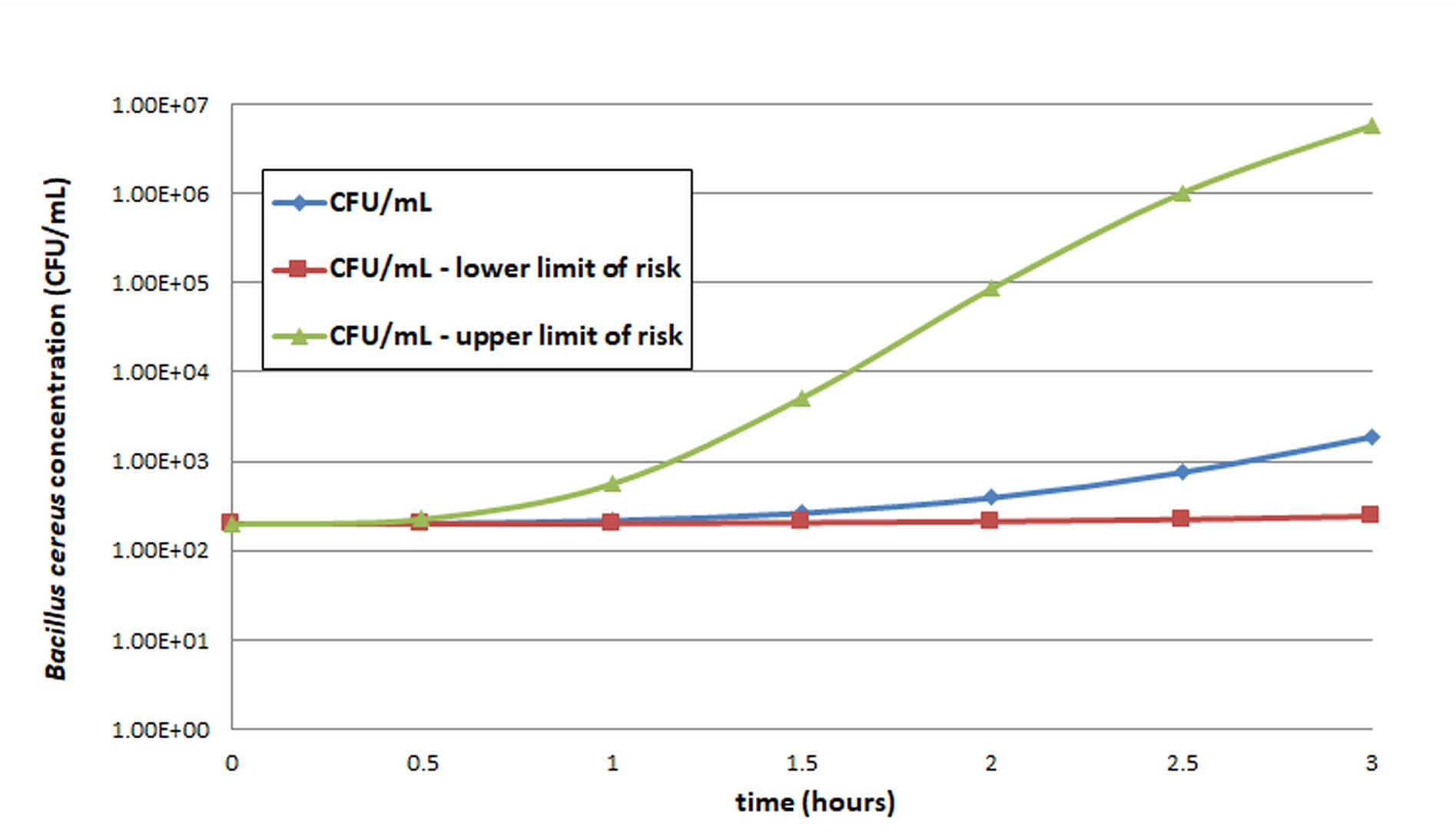

Supplement: Supplementary file 1 [file foods-11-01037-s001.zip › Figure S3_supplementary data.jpg]
